# Pulmonary Cystic Echinococcosis in Belgium

**DOI:** 10.7759/cureus.93389

**Published:** 2025-09-28

**Authors:** Alexander Ockerman, Philip Lerut, Stephanie Verschuere, Michaël Boudewijns

**Affiliations:** 1 Microbiology, AZ Groeninge Hospital, Kortrijk, BEL; 2 Thoracic Surgery, AZ Groeninge Hospital, Kortrijk, BEL; 3 Pathology, AZ Groeninge Hospital, Kortrijk, BEL

**Keywords:** cystic echinococcosis (ce), cystic lung lesion, echinococcus granulosus (e. granulosus), pulmonary cystic echinococcosis, thoracic surgery

## Abstract

Cystic echinococcosis (CE) is a zoonotic disease caused by the larval stage of the cestode *Echinococcus granulosus*. We report a case of a 28-year-old Belgian woman who presented with debilitating inspiratory dorsal pain and coughing. Imaging revealed a large cystic structure in the right lower pulmonary lobe. Following surgical resection, histopathological examination and molecular testing of the cyst confirmed infection with *E. granulosus* G1 genotype (sheep strain). The patient had no travel history outside of Europe but had professional exposure to dogs as a groomer and had adopted a dog from Morocco. Postoperative treatment with albendazole was administered for three months with a favorable clinical outcome, and to this day, there is no evidence of recurrence. This case highlights the importance of considering CE in the differential diagnosis of cystic lung lesions, even in non-endemic regions like Belgium.

## Introduction

Echinococcosis is a severe parasitic infection caused by the larval stages of cestodes of the genus *Echinococcus*. The two most common forms are caused by *E. granulosus* and *E. multilocularis*. They cause disease in both humans and animals and are two of the most widespread zoonoses of medical importance [1]. According to the World Health Organization (WHO), there may be more than 1 million people living with echinococcosis globally, with annual deaths estimated at around 20,000 [2]. Infection by *E. granulosus* is most frequent worldwide and causes cystic echinococcosis (CE), with formation of hydatid cysts in various organs. Infection by *E. multilocularis* causes alveolar echinococcosis (AE) with tumor-like lesions primarily in the liver and is endemic in Central Asia, Europe, and North America. "Neotropical echinococcosis," caused by *E. vogeli* and *E. oligarthrus,* remains limited to South America and is much rarer [3-5].

In Europe, there were 929 confirmed cases of human echinococcosis (incidence of 0.21 per 100,000) reported in 2023, with *E. granulosus* accounting for 67.3% and *E. multilocularis* for 32.7% of cases. This represents the highest number of confirmed human cases in the last 15 years in Europe [6]. This is possibly due to increased surveillance and reporting; nonetheless, an expansion of the European endemic area for *E. multilocularis* has been described [7]. 

In Belgium, there were 10 cases of AE and 20 cases of CE reported in 2023. All cases of AE were autochthonous Belgian infections, likely through contact with feces of infected foxes or dogs, with the majority of cases in Wallonia, where the infection rate of foxes by *E. multilocularis* is higher than in Flanders [8]. All cases of CE were infected outside of Belgium, as *E. granulosus* is not endemic in the country [9]. According to the Belgian National Reference Laboratory for Echinococcosis (CHU Liège), cases in Belgium have been increasing since 2010 [7,9]. Cases of isolated pulmonary CE are even rarer, with only two cases reported in 2023. This makes the diagnosis of pulmonary CE challenging in Belgium because it is rarely considered in the differential diagnosis of pulmonary cysts. 

Humans are accidental intermediate hosts of *Echinococcus* spp. They are most commonly infected through ingestion of *Echinococcus* eggs from soil, water, or food contaminated by dog or fox feces. Eggs can survive for at least one year in the environment [10]. After ingestion, eggs hatch in the small intestine, releasing hooked oncospheres which penetrate the intestinal wall and migrate through the circulatory system. In *E. granulosus*, cysts can develop in various organs, especially the liver (70%) and lungs (20%). In *E. multilocularis,* the liver is primarily affected. Here, the hydatid cysts develop, enlarging gradually over the years with compression or damage to surrounding tissues, producing protoscolices and daughter cysts that fill the cyst interior. In humans, cysts may rupture and create secondary cysts in other organs. Normally, the definitive host (dogs for *E. granulosus* and foxes for *E. multilocularis*) becomes infected by ingesting the cyst-containing organs of the infected intermediate host (usually herbivores such as sheep, cattle, or goats). The protoscolices in these cysts then attach to the intestinal mucosa, develop into the adult stage, release eggs into the feces, and the parasitic life cycle can start anew [3,11].

## Case presentation

**Figure 1:**
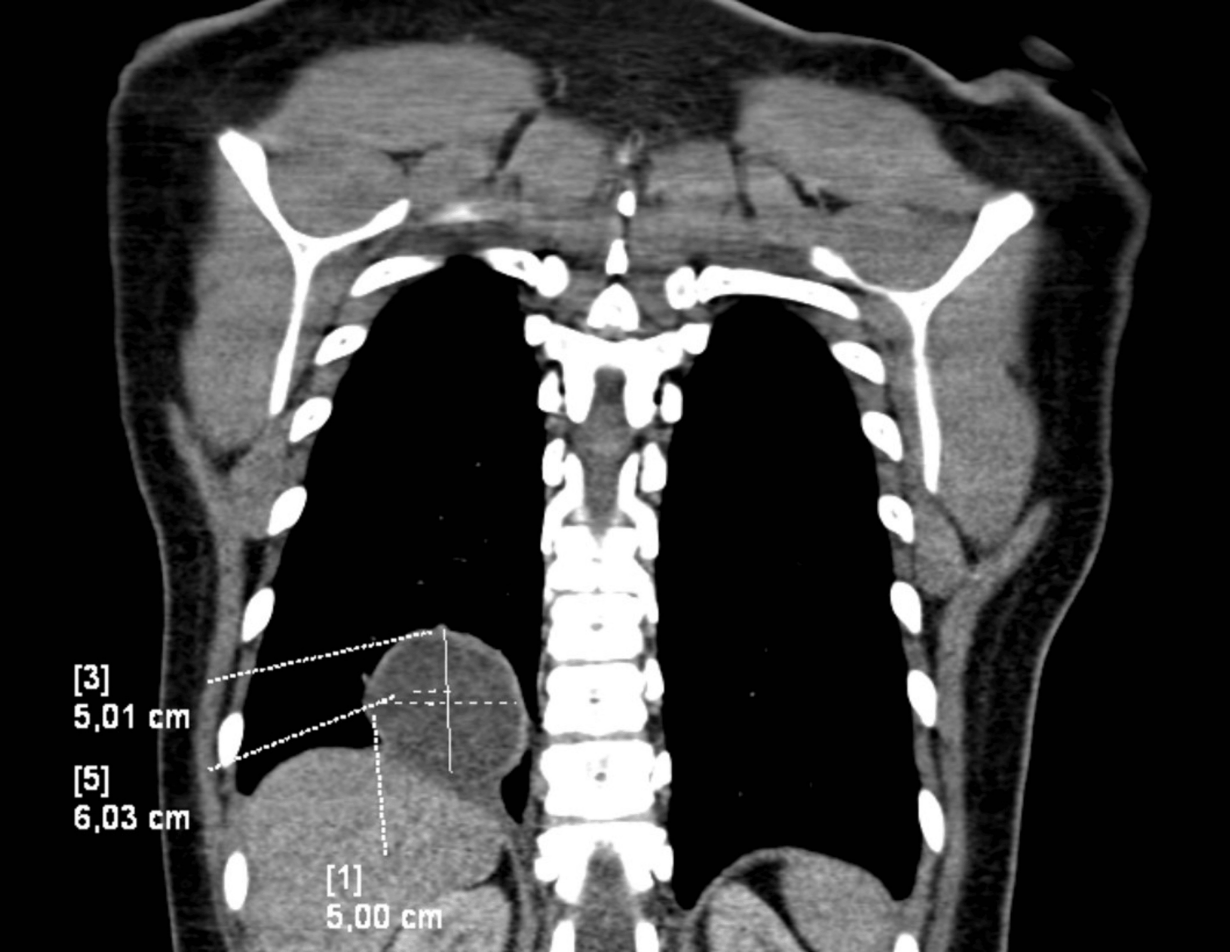
Coronal CT thorax image with contrast A large cystic structure was noted in the right lower pulmonary lobe on coronal CT thorax. The cyst has a well-circumscribed, regular wall and is filled with homogenic fluid.

A 28-year-old Belgian woman presented with debilitating inspiratory dorsal pain and coughing for one week. She was a non-smoker with an unremarkable medical history. A CT thorax scan showed a large cystic structure (5 cm × 6 cm × 6 cm) in the right lower pulmonary lobe (Figure 1). Routine blood analysis showed no abnormalities with low C-reactive protein levels (2.7 mg/L) and a normal eosinophil count (0.6%, 40/µL).

The patient was referred to the vascular surgeon, and a video-assisted thoracoscopic surgical wedge resection of the cyst was performed. An intraoperative frozen section showed a benign cyst wall with chronic inflammation. The postoperative course was without complications. Peroperative cultures of the cyst wall and fluid yielded no growth.

**Figure 2:**
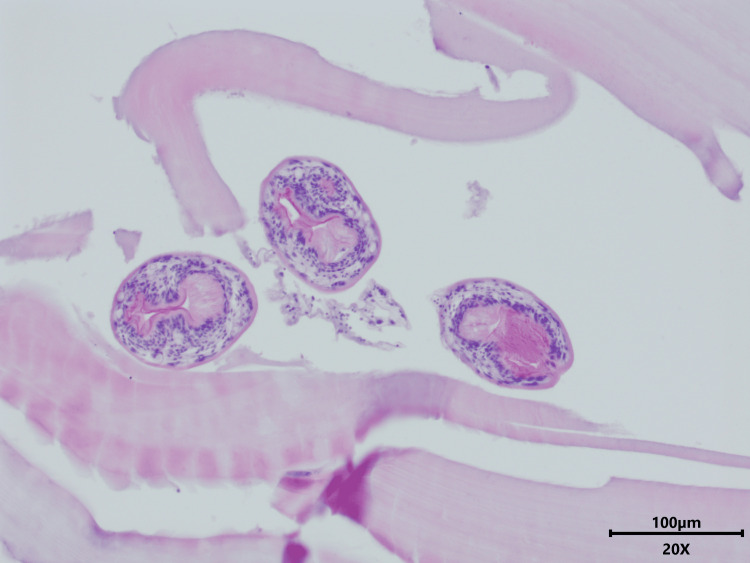
Protoscolices in hydatid cyst, hematoxylin & eosin stain (200× magnification) The histopathological examination of the cyst wall reveals an acellular laminated membrane with inflammatory cells on the inner surface. Within the lumen, multiple protoscolices are visible, which are characteristic of a hydatid cyst.

**Figure 3:**
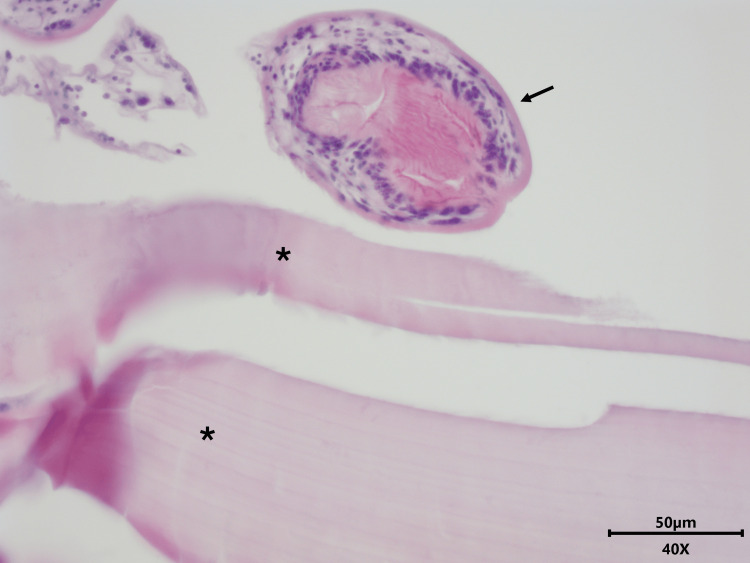
Detail of cyst wall and protoscolex, hematoxylin & eosin stain (400× magnification) The acellular laminated membrane is marked with an asterisk and the protoscolex is marked with an arrow.

**Figure 4:**
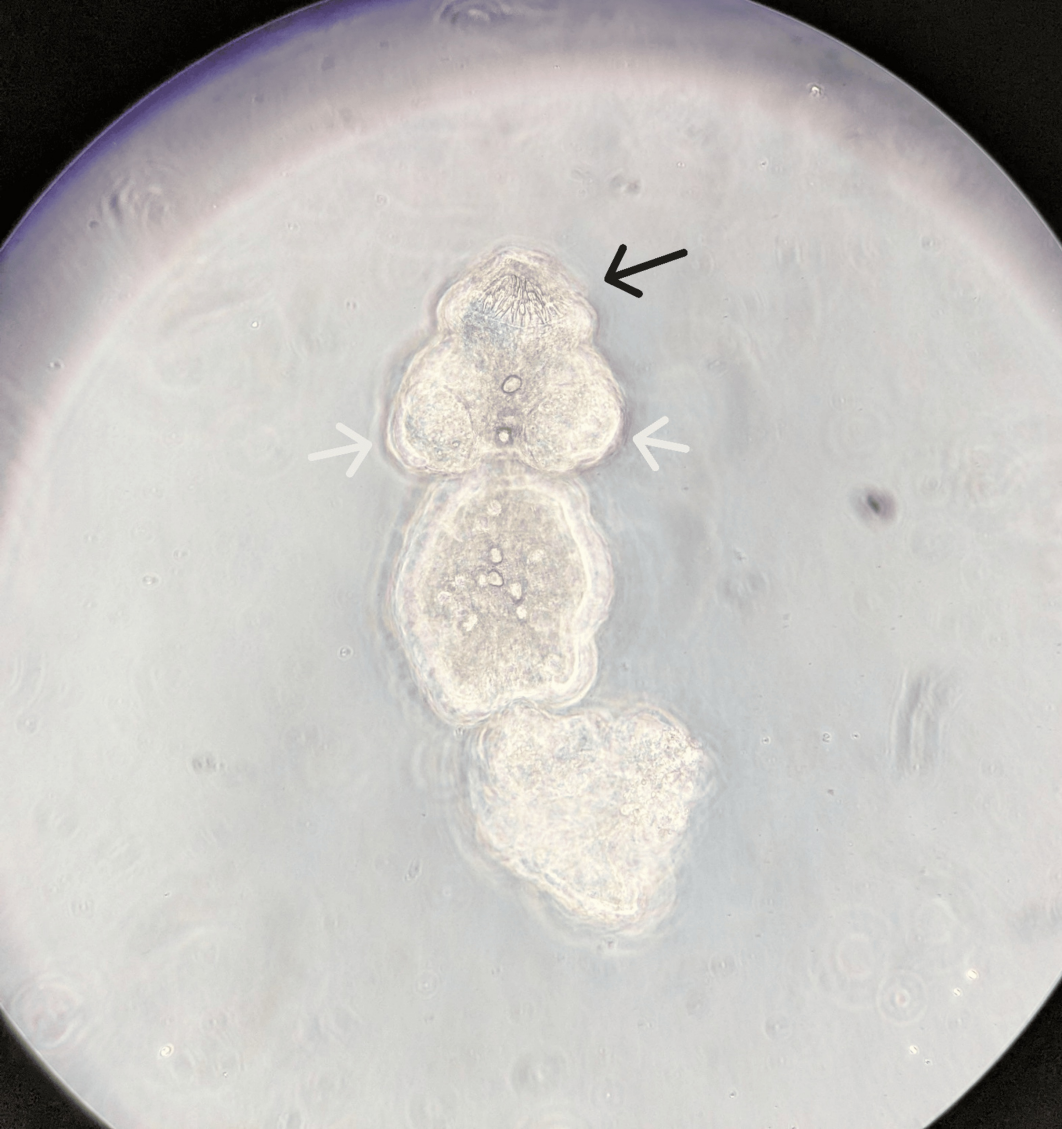
Protoscolex in hydatid cyst fluid, direct wet mount (100× magnification) A wet mount saline preparation of the cystic fluid showing an intact scolex with a clearly visible pair of suckers (white arrows) and ring of rostellar hooks (black arrow), which allow attachment to the intestinal wall of the definitive host.

Initially, there was no clinical suspicion of echinococcosis. Surprisingly, a few days later, the pathologist identified protoscolices in the cyst fluid, compatible with the diagnosis of a hydatid cyst (Figures 2, 3). This prompted the clinical laboratory to perform serology for *Echinococcus* species and PCR testing on the cyst fluid. Additionally, a wet mount of the fluid was evaluated microscopically, revealing a still motile scolex with clearly visible hooklets (Figure 4). Serology was negative for anti-*E. granulosus* and *E. multilocularis* antibodies (ELISA and hemagglutination assays). PCR on the cyst fluid was positive for *E. granulosus* (G1-profile sheep strain).

The patient had no history of travel outside of Europe. She did, however, have professional exposure to dogs as a dog groomer and had adopted a dog from Morocco several years ago. Since *E. granulosus* is not endemic in Belgium, the adopted Moroccan seems the most plausible source of infection for our patient. 

The patient was treated with albendazole 2 x 400 mg/d for three months after resection of the cyst. Liver enzymes were periodically monitored for albendazole toxicity. Additional imaging of the abdomen and brain did not show parasitic spread to other sites.

The clinical course was favorable, with total resolution of the respiratory complaints, and to this day, there is no evidence of recurrence on follow-up imaging four months after surgery. Yearly follow-up imaging will be provided. 

## Discussion

The *E. granulosis* G1 genotype (sheep strain), found in our case, is the most widespread genotype globally and is considered to be responsible for the vast majority of human and livestock CE cases worldwide [12]. The G1 genotype is associated with sheep as the intermediate host and is highly prevalent in Mediterranean countries, South America, and parts of Asia where sheep farming is common [13]. In Morocco, where our patient's adopted dog originated, CE is endemic with an estimated prevalence in sheep of 7-8%, making this a plausible chain of infection [14].

Serology, in our case, was negative. This is in line with the literature, which reports a low sensitivity (50-60%) of serologic assays for isolated pulmonary CE [15]. Encystment of the hydatid cyst with a thick collagen layer, which shields the cyst content from the immune system, can explain the often negative serology results. Positive serology is associated with complicated diseases like cyst rupture or surinfection, releasing parasitic antigens in the body and producing a serologic response [16].

Imaging techniques (X-ray or CT scan) are recommended by the WHO for screening and diagnosis of pulmonary CE, with serology now only used to confirm imaging results. 

There are no clinical trials that have compared diverse treatment approaches in pulmonary CE. Surgical removal of the cyst is considered the cornerstone of treatment when feasible, followed by systemic antiparasitic therapy. Albendazole is considered the first-choice antiparasitic agent. It can also be used preoperatively to minimize the risk of recurrence due to peroperative spilling of protoscolices-rich cystic fluid or as definitive therapy when surgery is contraindicated [3,17]. There is, however, no consensus on treatment length and schedules.

In this case, there was no prior suspicion of pulmonary echinococcosis, so no preoperative albendazole was administered. Postoperatively, the patient was treated with albendazole 400 mg 2×/d for three months (Belgian Society for Infectiology and Clinical Microbiology guidelines).

## Conclusions

While echinococcosis remains a rare disease in Belgium, cases are on the rise. The case presented here illustrates the importance of considering CE in the differential diagnosis of cystic lung lesions, even in patients without any travel history to endemic regions. Medical practitioners in Belgium should be increasingly aware of echinococcosis, particularly in patients with risk factors such as contact with dogs from endemic regions. Increased surveillance and reporting systems are important for monitoring the epidemiological trends of this important zoonotic disease.
